# Ag nanorod@PEI-Ag nanohybrid as an excellent signal label for sensitive and rapid detection of serum HER2

**DOI:** 10.1038/s41598-023-48838-3

**Published:** 2023-12-08

**Authors:** Fatemeh Hakimian, Mohammad Mazloum-Ardakani

**Affiliations:** Department of Chemistry, Faculty of Science, Yazd University, Yazd, 89195-741 Iran

**Keywords:** Analytical chemistry, Electrochemistry, Biochemistry, Chemistry

## Abstract

The accurate detection of Human epidermal growth factor receptor-2 (HER2) as a critical breast cancer biomarker can be essential for the early selection of therapeutic approaches. HER2 is a prominent component of a signaling network. Overexpression of the HER2 protein due to amplification of its gene leads to the development of an aggressive subtype of breast cancer. Patients with tumors that overexpress HER2 are eligible for treatment that significantly reduces mortality rates. Herein, we present a fast and simple method for detecting serum HER2. A new electrochemical label has been developed using charged Ag nanorod@ polyethylenimine-Ag (Ag NR@ PEI-Ag) nanohybrid. The synthesized Ag NR@PEI-Ag nanohybrid simultaneously has the electroactive property of silver and the large surface area of the PEI, which results in the enhancement of the detection signal. So, using Ag NR@PEI-Ag nanohybrid as the electrochemical label, a simple, fast, and sensitive electrochemical biosensor was designed to detect HER2. This way, after immobilizing HER2 aptamer on the Au electrode surface, HER2 or human serum was exposed to the aptamer. Then, the positively charged Ag NR@PEI-Ag nanohybrid was adsorbed onto the negatively charged aptamer-HER2 complex, and the current that was produced due to the Ag/AgCl reaction was measured as the electrochemical signal. The aptasensor shows a broad linear response from 10^–12^ to 10^–7^ g, a low detection limit (LOD) of 10 pg, and a total assay time of ~ 30 min.

## Introduction

According to the statistics, in 2020, 2.3 million women worldwide were diagnosed with breast cancer, and unfortunately, 685,000 people died^1^. Late screening is one of the leading causes of this deadly disease^2^. Early detection of cancer and effective treatment planning can significantly increase survival rates.

In this regard, there are various diagnostic methods for breast cancer detection, including biopsy^3^, breast magnetic resonance imaging (MRI)^4^, and mammography^5,6^. Although these methods are sensitive, they have some disadvantages. For example, the biopsy is highly invasive and allows only a small part of a tissue to be analyzed. MRI and mammography also have disadvantages, such as the requirement for sophisticated equipment and inducing radiation doses in sensitive tissue (breast)^7,8^. One solution is the analysis of cancer biomarkers found in physiological fluids such as blood^9–12^.

The primary breast cancer-related protein biomarkers for non-invasive clinical tests are the Human Epidermal growth factor Receptor 2 (HER2 or ErbB2), Carcinoembryonic Antigen (CEA), and Cancer Antigen 15–3 (CA15-3)^13–15^. HER2, as a critical prognostic indicator^16^, is overexpressed in 20 to 30% of breast cancers known as HER2-positive breast cancers^17^. HER2 stimulates the breast cancer cells to grow and spread more aggressively^18^. Sensitive detection of HER2 in serum samples is clinically essential for early awareness and breast cancer treatment^19^. At the moment, diagnostic tests of HER2 are mainly antibody-based methods like enzyme-linked immunosorbent assay (ELISA)^18^ and electrochemical immunosensing^20,21^. Despite the advantages of these methods, their most significant drawback is that antibodies are expensive and challenging to modify^10^.

Aptamers are short, single-stranded RNA or DNA molecules^22^ with an inexpensive and specific binding ability with a particular target, such as small molecules^23^, proteins^24^, and whole cells^25^. They are widely used to design biosensors because of their easy compatibility with various platforms^26^. Aptasensors are a class of biosensors that use a DNA or RNA aptamer as the biological recognition element^27^. Aptasensors can be classified based on their transducer types, such as electrochemical, optical, mass-sensitive, or thermal.

Among those conventional detection techniques, electrochemical aptasensors have attracted considerable attention for the detection of biomarkers due to their analytical characteristics, including high sensitivity, simplicity, fast, low cost, and real-time detection^26,28–30^.

Nanomaterials are widely used to construct biosensors to enhance their sensitivity^31–37^. Some recent nanomaterials that have enhanced the performance of biosensors include WS2 nanoflowers on N, B-doped graphene for early detection of Phenylketonuria^38^, Fe_3_O_4_NPs@covalent organic framework decorated gold nanoparticles for sensing of α-fetoprotein^39^, nanocomposite of multiwalled carbon nanotubes/molybdenum disulfide nanoparticles for reliable detection of paraoxon^40^, CdMoO_4_/g-C_3_N4 nanocomposite for carbendazim determination^41^, and manganese sulfide nanoparticles/graphene oxide/polyaniline for usage in tau protein detection^42^.

As widely used nanomaterials, metal nanoparticles comprise thousands of atoms that can be electrochemically oxidized or reduced. Therefore, they can provide significant signal amplification of the transduction signal of a detection event, which causes a noticeable increase in the sensitivity and performance of electrochemical biosensors. Silver nanoparticles, one of the most widely used metal nanoparticles, have high conductivity, significant electrochemical reactivity, sharp oxidation peaks, and low redox potentials^43,44^.

In this work, for the first time, we synthesized Ag nanorod@ polyethylenimine-Ag (Ag NR@PEI-Ag) nanohybrid in which PEI is considered a reductant, stabilizer, and a factor for surface area enhancement. Then, using Ag NR@PEI-Ag nanohybrid as a great electrochemical label, an ultrasensitive aptasensor for detecting HER2 protein in human serum was designed. This fast, simple, and sensitive electrochemical method can positively impact the early diagnosis of breast cancer.

## Results and discussion

### Characterization of Ag NR@PEI-Ag nanohybrid

**Figure 1:**
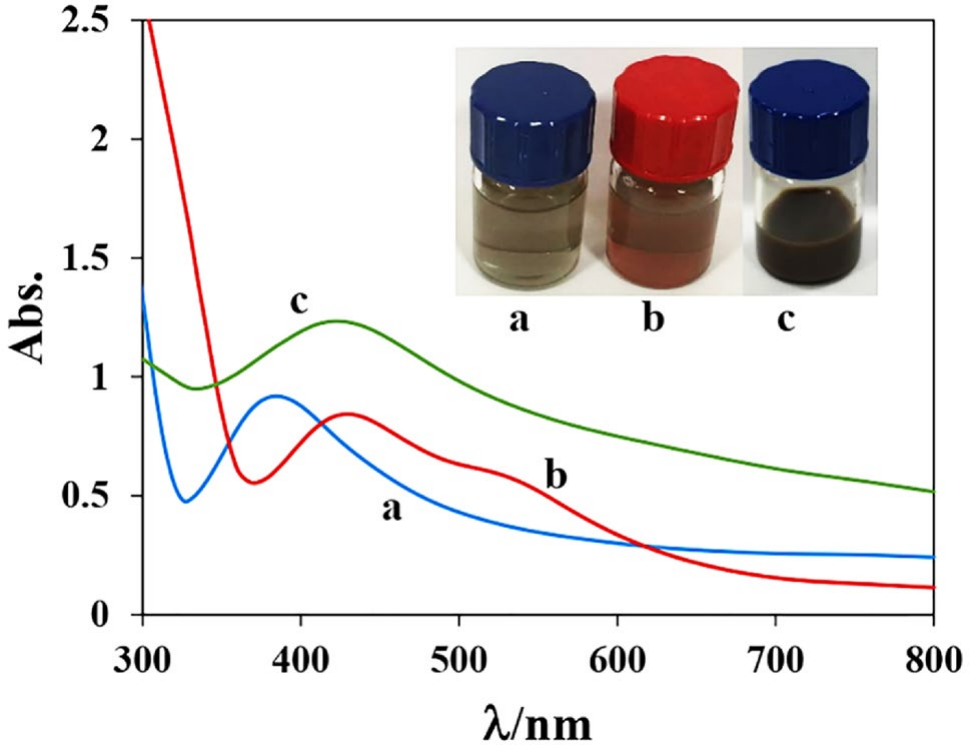
UV–Vis absorption spectra of (**a**) seed, (**b**) Ag NR, and (**c**) Ag NR@PEI-Ag. The inset (from **a** to **c**) shows photographs of the color-changing in sample solutions.

Various methods were used to confirm the formation of Ag NR@PEI-Ag nanohybrid. Figure 1 compares the UV–Vis absorption spectra of seed, Ag NR, and Ag NR@PEI-Ag nanohybrid. The seed shows a single absorption peak at ~ 400 nm, the characteristic peak attributed to the surface plasmon resonance (SPR) band of silver nanoparticles^45^. As seen in Fig. 1b, Ag NRs show two plasmon absorption bands at 435 and 540 nm, indicating the successful formation of Ag NR. It is worth mentioning that the oscillation of electrons on the surface of Ag NRs has two oscillation modes: transverse and longitudinal plasmon oscillation. Transverse oscillation, which is mainly correlated to the diameters of Ag NRs, appears at the shorter wavelength side (~ 400 nm). On the other hand, the longitudinal plasmon resonance is related to the length of Ag NRs. So, the absorption peak of Ag NR's length emerges at the longer wavelength side (˃ 500 nm)^46,47^.

Interestingly, by forming an Ag NR@PEI-Ag nanohybrid, the two SPR peaks of Ag NR are changed to one peak at 425 nm. As seen in Fig. 1c, the SPR band of Ag NR@PEI-Ag shows a blue shift while peak broadening occurs, probably due to Ag NR's coverage with the PEI-Ag hybrid. The corresponding photo images of the seed, Ag NR, and Ag NR@PEI-Ag nanohybrid were shown in the inset of Fig. 1. As seen, the seed is green, which turns orange and green-gray with the formation of Ag NR and Ag NR@PEI-Ag nanohybrid, respectively.

**Figure 2:**
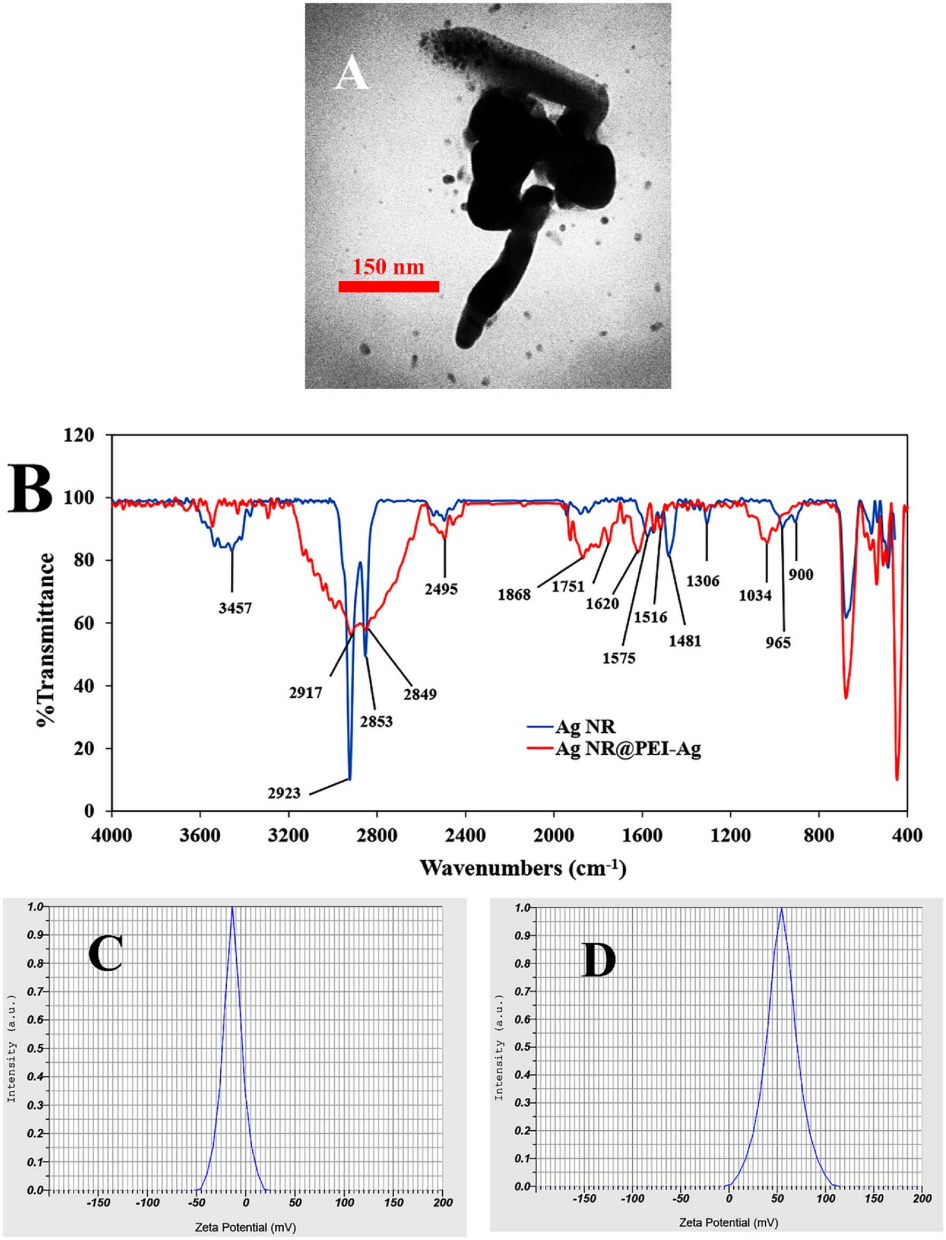
(**A**) TEM image of Ag NR@PEI-Ag, (**B**) FTIR spectra; (**C** and **D**) zeta potential of Ag NR and Ag NR@PEI-Ag.

The morphology of the Ag NR@PEI-Ag nanohybrid was analyzed by TEM. As seen in Fig. 2A, the formed nanoparticles are mostly rod-like. However, some spherical nanoparticles are also formed. Further information regarding the chemical bonding structure and the changing surface species was obtained from FTIR spectroscopy. Figure 2B compares the FTIR spectrum of Ag NR and Ag NR@PEI-Ag nanohybrids. The FTIR spectrum of Ag NR shows a wide absorption band at 3457 cm^−1^, representing the OH groups' stretching frequency^48^. Two sharp peaks at 2923 and 2853 cm^−1^ can correspond to asymmetric and symmetric stretching of CH of the alkyl chain in CTAB^49^. The bands at 1575 and 1481 cm^−1^ can be assigned to asymmetric and symmetric C-H scissoring vibrations of the CH_3_-N^+^ moiety of CTAB. In the FTIR spectrum of Ag NR@PEI-Ag nanohybrid, the peaks at 1575 and 1481 cm^−1^ broaden and shift to 1868 and 1620 cm^−1^ sequentially, indicating that capping of PEI-Ag on Ag NR (which is covered with CTAB) occurs via head groups of CTAB^49^. The asymmetric and symmetric CH_2_ stretching vibrational frequencies are observed at 2917 and 2849 cm^−1^ sequentially^50^, which is broad compared to the Ag NR. The band that appears at 2495 cm^−1^ is the result of isotopic exchange in some OH groups^51^. The peak at 1516 cm^−1^ is likely because of C–N stretching and N–H bending of PEI^52^. The peak at 1034 cm^−1^ is attributed to the C–N stretching mode^50^.

DLS measured the zeta potential of Ag NR and Ag NR@PEI-Ag nanohybrid to be − 14.1 ± 0.78 mV and 55 ± 0.92 mV, respectively (Fig. 2C and D). The positive zeta potential of Ag NR@PEI-Ag nanohybrid is due to the amine groups of PEI.

**Figure 3:**
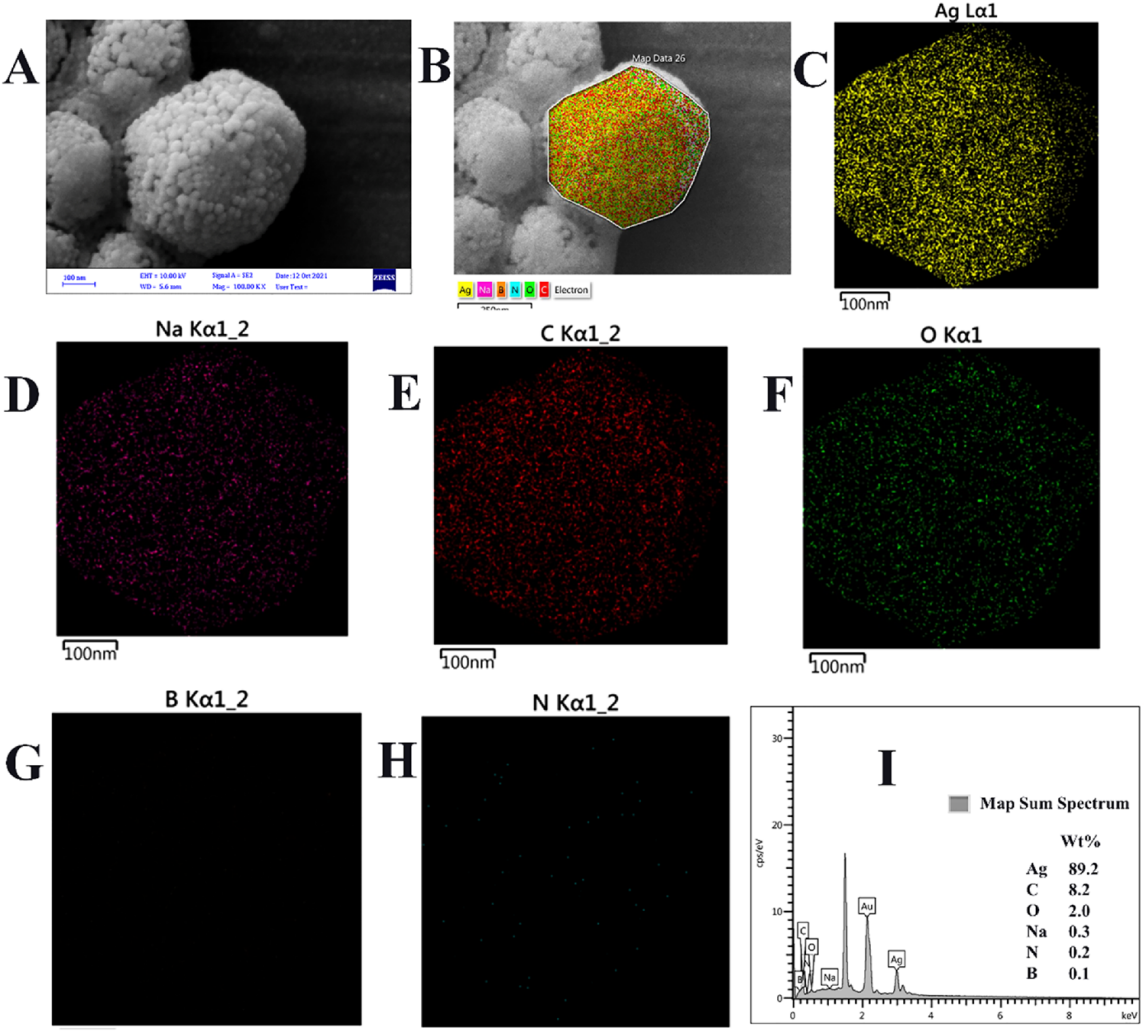
SEM image (**A**); elemental X-ray mapping (from **B** to **H**) and EDX analysis (**I**) of the area marked with a multifaceted (in **B**); of the as-prepared Ag NR@PEI-Ag nanohybrid.

To investigate the compositional analysis and distribution of major elements of the Ag NR@PEI-Ag nanohybrid, elemental mapping, and EDX line scans were obtained from the Ag NR@PEI-Ag nanohybrid. Figure 3 shows the resulting compositional images where areas investigated for EDX analysis are marked with a multifaceted in Fig. 3B. The results confirmed the presence of Ag, Na, C, O, B, and N in the Ag NR@PEI-Ag nanohybrid. Ag, Na, C, O, B, and N are presented in yellow, pink, red, green, orange, and cyan, respectively. The Ag peak originates from the Ag shell. C and N peaks are attributed to the PEI, which comprises amine groups and carbon aliphatic CH_2_CH_2_ spacers. The presence of Na, B, and O peaks is probably due to the ions in the solution. Elemental mapping by EDX (Fig. 3) confirms the uniform distribution of elements.

### Detection mechanism

**Figure 4:**
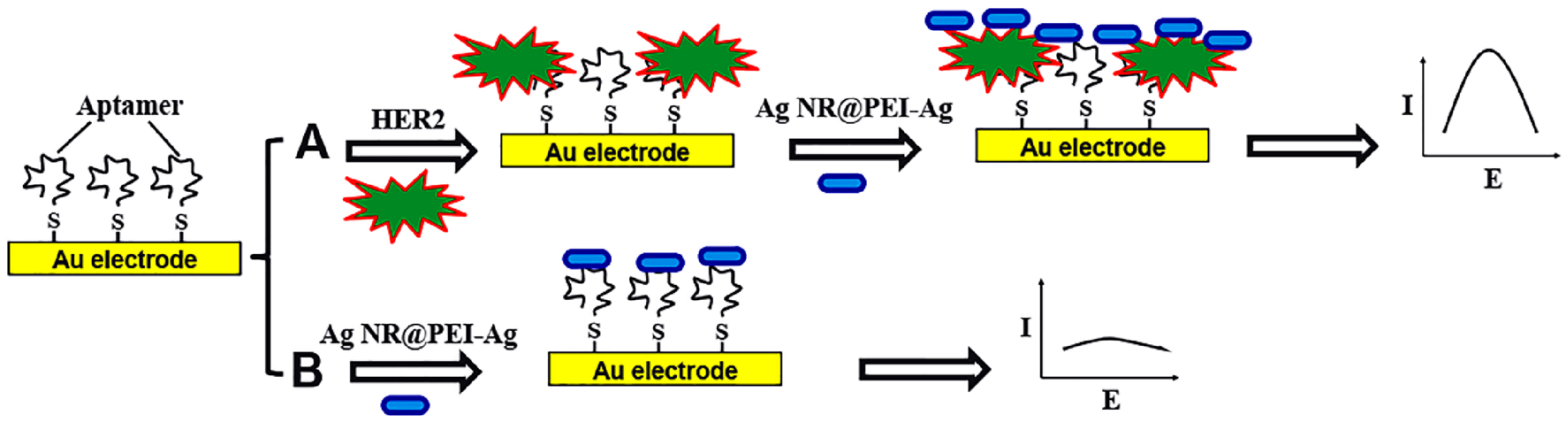
Schematic illustration of the fabrication of the electrochemical aptasensor.

The fabrication process of the aptasensor is summarized in Fig. 4. First, the thiolated aptamer was covalently attached to the surface of the Au electrode. Then, the target (HER2) was introduced into the electrode, and afterward, the Ag NR@PEI-Ag nanohybrid was cast on the modified Au electrode. If the target (HER2) exists in the environment, the aptamer/HER2 complex is formed. The aptamer/HER2 complex has a more negative charge than aptamer alone (in the absence of HER2), which causes a more positively charged Ag NR@PEI-Ag nanohybrid to accumulate on the electrode, resulting in a stronger current. For further explanation, it should be noted that the aptamer charge due to the phosphate groups in its backbone is negative. By complex formation, the negative charge density on the electrode surface due to the negative charge of HER2 is increased.

On the other hand, Ag NR@PEI-Ag nanohybrid has a very positive charge due to the nitrogen atoms in PEI branches. So, positively charged Ag NRs@PEI-Ag nanohybrid can bind the aptamer/HER2 complex electrostatically more than those to the aptamer alone, which has a less negative charge (Fig. 4, path A). Without HER2, there is less electrostatic attraction between the aptamer alone and Ag NRs@PEI-Ag nanohybrid (Fig. 4, path B). Consequently, in the presence of HER2, higher electrostatic absorption of Ag NRs@PEI-Ag nanohybrid results in a more robust electrochemical response.

Because even in the absence of HER2, a limited electrostatic attraction occurs between negatively-charged aptamer alone and positively-charged Ag NR@ PEI-Ag nanohybrid (Fig. 4, path B), a background current is observed in all electrochemical measurements. Besides, any changes in the aptamer's immobilization may result in changes in this background current. So, these changes in responses reduce the reproducibility of the result of the aptasensor. To remove the effect of background current and to replicate the results further, relative current response (δI) was used as the response of the aptasensor^53–55^ according to Eq. (1).

1 $${\delta I} = \left| {\frac{{I_{2} - I_{1} }}{{I_{2} }}} \right|$$

I_2_ and I_1_ show the amount of current generated sequentially in the presence and absence of HER2. It should be noted that all-electric current responses (I_1_ and I_2_) are recorded in the presence of Ag NRs@PEI-Ag nanohybrid. In Eq. (1), the higher the value of δI, the higher the accumulation of Ag NRs@PEI-Ag nanohybrid on the modified electrode due to the higher concentration of HER2 in the medium. The concentration of HER2 in the sample determines the likelihood of aptamer/HER2 complex formation. Thus, the further negative charge of the aptamer/HER2 complex boosts the probability of Ag NRs@PEI-Ag nanohybrid binding via electrostatic interactions.

### Optimization of experimental parameters

**Figure 5:**
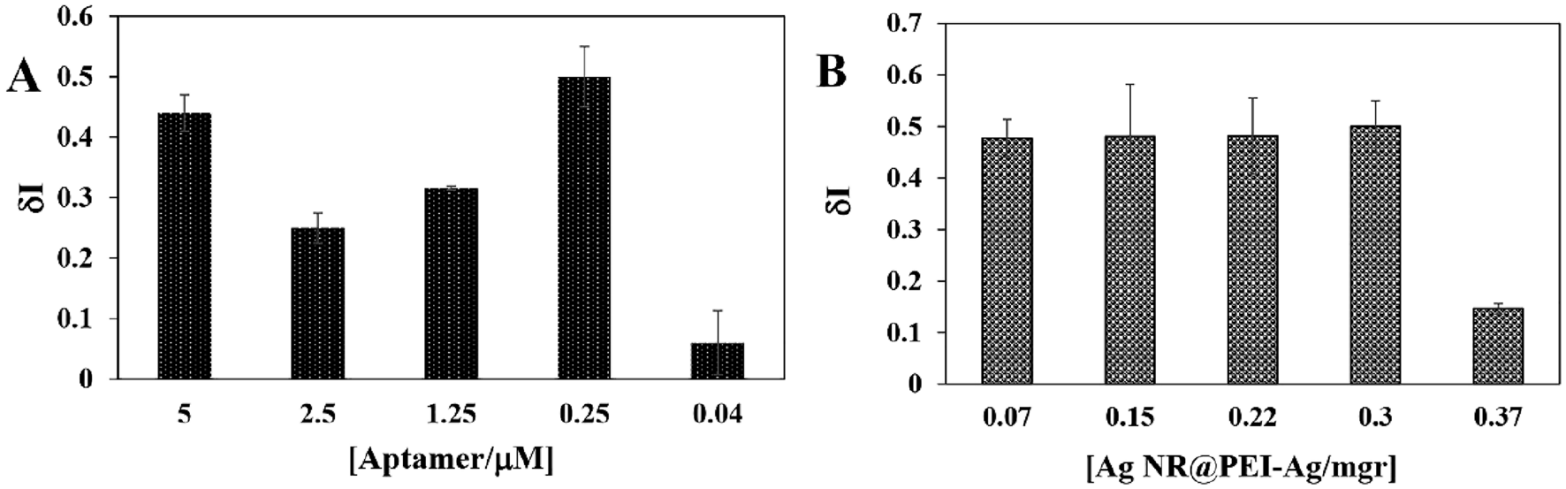
Optimization of aptamer concentration (**A**) and the amount of Ag NR@PEI-Ag nanohybrid (**B**) for the fabricated aptasensor. Experiments were performed in 0.1 M PBS (pH 7) and at a scan rate of 100 mV/s.

The effect of the aptamer concentration loaded on the Au electrode (AuE) was investigated over the response of the aptasensor. For this purpose, various aptamer concentrations (0.04, 0.25, 1.25, 2.5, and 5 µM) were loaded on the AuE surface. After the addition of HER2 to each concentration of the aptamer, the CVs were measured in the presence of Ag NRs@PEI-Ag nanohybrid (0.3 mg). As shown in Fig. 5, the optimum amount of aptamer was selected to be 0.25 µM.

For optimization of Ag NR@PEI-Ag nanohybrid concentration, different amounts of Ag NR@PEI-Ag nanohybrid (0.07, 0.15, 0.22, 0.3, and 0.37 mg) were cast on the AuE/Aptamer and AuE/Aptamer/HER2 for each concentration of Ag NR@PEI-Ag. Then, the CVs were recorded, and δI was calculated. As shown in Fig. 5, the best δI was obtained for Ag NR@PEI-Ag nanohybrid at a concentration of 0.3 mg. It should be noted that subsequent electrochemical measurements were performed concerning the optimal concentrations of Ag NR@PEI-Ag nanohybrid and aptamer.

### Analytical approach of HER2 aptasensor

**Figure 6:**
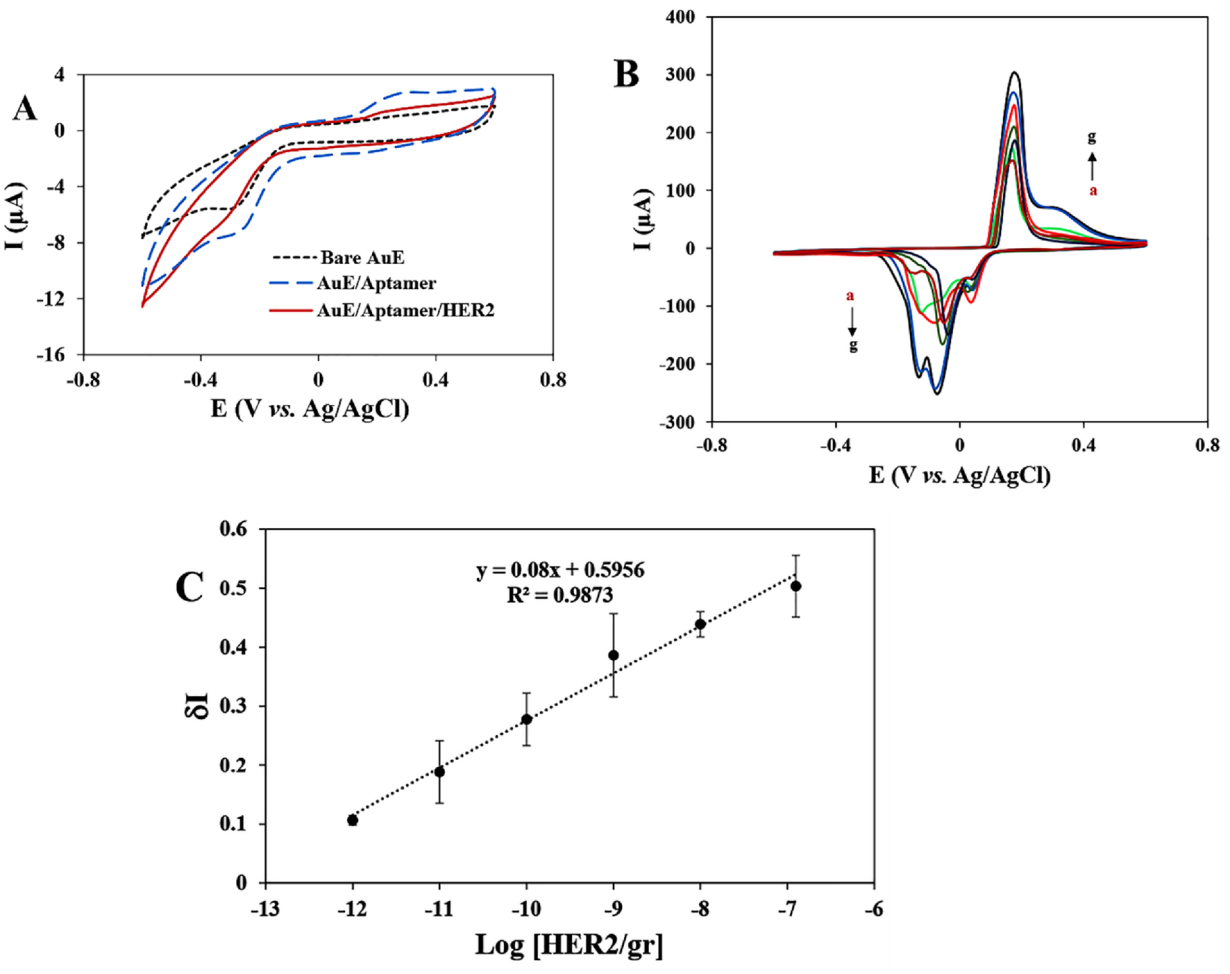
(**A**) The CVs of a gold electrode (AuE) before and after immobilization of aptamer and HER2. (**B**) The CVs of the AuE-aptamer after casting Ag NR@PEI-Ag in the presence of different concentrations of HER2: a: 0, b: 10^−12^, c: 10^−11^, d: 10^−10^, e: 10^−9^, f: 10^−8^, and g: 10^−7^ gr. CVs were recorded in 0.1 M PBS at a scan rate of 100 mV s^−1^. C) Calibration plot for determination of HER2.

In Fig. 6A, the CVs obtained for different stages of modification in the absence of Ag NR@PEI-Ag nanohybrid in the 0.1 M PBS solution (pH 7) at the scan rate of 100 mV s^−1^ are compared. The voltammogram intensity of the aptamer-modified Au electrode (AuE/Aptamer) is slightly higher than that of the bare Au electrode (bare AuE) due to the formation of more oxides on the electrode surface. This phenomenon can be due to the electrostatic adsorption of most metal ions by negatively charged aptamer^56^. By adding HER2 to the electrode surface, the peak intensity is reduced. Although there is more negative charge near the electrode surface, the Aptamer/HER2 modified Au electrode (AuE/Aptamer/HER2) creates more spatial barrier and less surface space for metal ions to accumulate on the electrode surface, which is why the peak intensity decreases^57^. Therefore, the peak intensity change confirms the electrode surface's modifications.

The Fig. 6B shows the cyclic voltammograms of aptamer and aptamer/HER2 (at different concentrations) in the presence of Ag NR@PEI-Ag nanohybrid in the 0.1 M PBS (pH 7) at the scan rate of 100 mV s^−1^. The electrode in the presence of Ag NR@PEI-Ag shows a very significant redox current versus Ag/AgCl reference electrode. The cathodic and anodic peaks are sequentially at the potentials of − 0.01 and 0.2 V, which correspond to the redox reaction (b).

2 $$Ag + Cl^{ - } \rightleftharpoons AgCl + e^{ - }$$

It should be noted that in the synthesis of the PEI-Ag hybrid on the Ag NR core, PEI acts as the reducing agent to reduce Ag^+^ to Ag^0^ (Ag nanoparticles). In accordance with reaction (Eq. 2), at the surface of the electrode, the formed Ag nanoparticles on PEI branches are oxidized to AgCl in the presence of NaCl in PBS. This electrochemical process can be the primary cause of the sharp redox peak generated by the aptasensor (Fig. 6B). In addition, the large surface area of PEI branches is the main reason for the transferring of electrons during the redox reaction (Eq. 2)^58,59^.

It should be noted that in some cases, splitting may happen in peaks like the anodic peaks in Fig. 6B. This phenomenon is probably due to the existence of a heterogeneous electrode, which results in a heterogeneous electron-transferring process^60^. Moreover, the formation of some species, such as silver oxide (Ag_2_O) and silver hydroxide (AgOH), can also cause peak splitting^61^.

To appraise the analytical performance of the aptasensor, different concentrations of HER2 were detected under optimal experimental conditions. Figure 6B shows that the CV response is gradually enhanced with the increase of the HER2 concentration. It is necessary to mention that due to the current reproducibility, only cathodic peak currents have been used in this work. After measuring the aptasensor CVs for a wide range of HER2 concentrations, a calibration curve of δI against the HER2 concentration logarithm was drawn. As shown in Fig. 6C, the linear range of the aptasensor for HER2 is from 10^−12^ to 10^−7^ g. The detection limit (LOD) of the aptasensor is 10 pg based^62,63^ on Eq. (3), where Sb and SDb are the average value and standard deviation of the blank (control) signals, respectively.

3 $$LOD = Sb + 3 \times SDb$$

To further explore the developed electrochemical aptasensor, a comparison of the method's performance with other methods is summarized in Table 1. The results confirm that the designed aptasensor performs better than other sensors.

**Table 1:** Comparison of recent HER2 electrochemical biosensors and the aptasensor developed in this work.

Materials	Technique	Linear range	Detection limit	Ref
Au electrode (AuE)/aptamer/HER2/Ag NR@PEI-Ag nanohybrid	CV	1–10^5^ pg	10 pg	This work
Screen-printed carbon electrode (SPCE)/poly-L-lysine/aptamer/HER2/MB	DPV	10–60 ng/mL	3 ng/mL	^28^
AuSPE/phenol-HER2/SDS-acetic acid	CV/DPV/EIS	10–70 ng/mL	1.6 ng/L	^64^
SPCE/ magnetic beads/Ab/BSA/HER2/Biotinylated Ab/Alkaline phosphate/Silver ions	LSV	5–50 and 50–100 ng/mL	2.8 ng/mL	^65^
AuE/AuNP/3-mercaptopropionic acid/aptamer/BSA/HER2	EIS	10^−5^–10^2^ ng/mL	5 ng/mL	^27^
Graphite electrode/reduced graphene oxide nano‑sheets/Rh-NPs/aptamer/HER2	DPV	10–500 ng/mL	0.667 ng/mL	^66^
AuE/aptamer/PEG/MB/HER2	CV	10^−12^–10^−8^ M	10^−12^ M	^67^
Interdigitated AuE/Thiol terminated aptamer/Saline-Tween 20/HER2	EIS	0.1–10^4^ ng/mL	0.1 ng/mL	^68^
Multiwall carbon nanotube-ionic liquid electrode/AuNP/1,6-hexane dithiol/AuNP/Ab/HER2	CV/EIS	10–110 ng/mL	7.4 ng/mL	^69^

### Selectivity and real sample analysis

**Figure 7:**
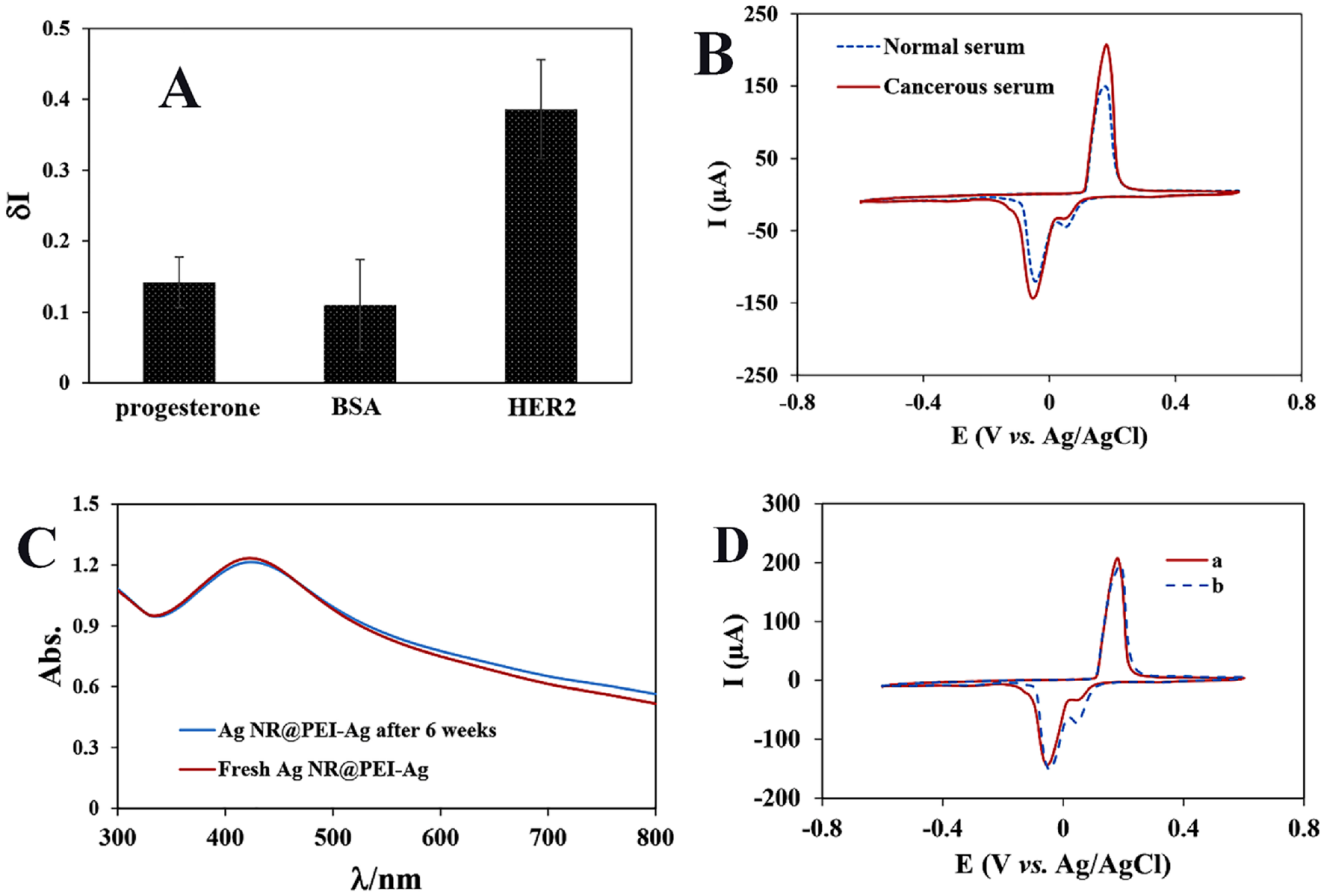
The specificity of the fabricated aptasensor to HER2 (**A**). Aptasensor responses to human serums including cancerous and normal (**B**). Storage stability of Ag NR@PEI-Ag nanohybrid and the modified gold electrode: (**C**) UV–Vis spectra of Ag NR@PEI-Ag nanohybrid immediately after synthesis and 6 weeks later; (**D**) The aptasensor response in the presence of HER2 immediately after immobilization of aptamer (a), and 10 days later (b).

To study the selectivity of the aptasensor, δI of progesterone, BSA, and HER2 were compared. As shown in Fig. 7A, a significant signal change was just observed for HER2, confirming the high selectivity of the aptasensor.

To demonstrate the accuracy of the aptasensor in human samples, human clinical samples containing normal and cancerous serums were prepared from Shahid Sadoughi Hospital (Yazd, Iran) and tested using the aptasensor. It is worth mentioning that before use, the sera were diluted with PBS buffer (0.1 M) in a ratio of 1:1. As shown in Fig. 7B, the presence of cancerous serum produces a higher current than normal serum, indicating a successful and simple differentiation between healthy individuals and cancer patients.

### Stability of the aptasensor

To evaluate the stability of Ag NR@PEI-Ag nanohybrid as an electrochemical label, UV–Vis spectra of Ag NR@PEI-Ag nanohybrid were recorded immediately after synthesis and 6 weeks later at room temperature (Fig. 7C). As can be seen, after 6 weeks, the spectrum has not changed significantly, indicating the stability of Ag NR@PEI-Ag for at least 6 weeks at room temperature. In addition, the stability of the modified electrode after 10 days was investigated. As shown in Fig. 7D, the CV signals indicate that the modified electrodes have acceptable stability after 10 days of storage at 4 °C.

In summary, we first successfully synthesized Ag NR@PEI-Ag nanohybrid as a good electrochemical label. Then, using an as-prepared Ag NR@PEI-Ag nanohybrid, a simple and sensitive electrochemical aptasensor was introduced for the specific detection of biomarker HER2. Ag NR@PEI-Ag nanohybrid, due to the excellent electroactivity of silver metal and the high surface area of PEI, significantly amplifies the electrochemical signal. The constructed aptasensor shows a wide linear range, low LOD, and outstanding capability to diagnose healthy and cancerous serums. The results show that the aptasensor can be used in clinical point-of-care applications for breast cancer screening. Also, this method has great potential to build different aptasensors for the sensitive and convenient detection of different biomarkers.

## Methods

### Materials and instruments

Silver nitrate (AgNO_3_), trisodium citrate, ascorbic acid, sodium hydroxide (NaOH), H_2_SO_4_, NaH_2_PO_4_, Na_2_HPO_4_, KCl, bovine serum albumin (BSA), and aluminum oxide were purchased from Merck (Germany). NaBH4 was obtained from Fluka (Switzerland). Cetyltrimethylammonium bromide (CTAB), HER2 (GST-tagged, human), H_2_O_2_, KOH, progesterone, and polyethylenimine (50% solution, Mn ~ 1200, Mw ~ 1300) were purchased from Sigma-Aldrich (USA). The HPLC purified HER2 aptamer sequence is as follows: 5’-SH GGGCCGTCGAACACGAGCATGGTGCGTGGACCTAGGATGACCTGAGTACTGTCC-3' was purchased from Bioneer Corporation (Republic of South Korea). The water used in the experiments was double-distilled.

UV–Vis absorption spectra of the synthesized particles were measured using an Optizen 3220 UV − Vis spectrophotometer. Fourier Transform Infrared (FTIR) spectral analyses were performed on a Nicolet iS50 FTIR spectrometer (Thermo Scientific, USA). An SZ-100 Nanoparticle Analyzer from Horiba-Jobin Yvon (Horiba, Ltd., Kyoto, Japan) was used for zeta potential measurements. EDAX and elemental distribution mapping were carried out on a Quanta 200 Scanning Electron Microscope (SEM). The transmission electron micrograph (TEM) image was recorded with a Philips EM208S microscope. Electrochemical measurements were carried out using Autolab potentiostat/galvanostat (PGSTAT-302 N, Netherlands).

All electrochemical experiments were performed at room temperature in a typical three-electrode system consisting of a platinum wire as a counter electrode, a modified gold as the working electrode, and an Ag/AgCl as a reference electrode (from Azar Electrode, Iran). Cyclic voltammetry (CV) was carried out in the presence of phosphate buffer saline (PBS 0.1 M, pH = 7) and the potential range of − 0.6 to 0.6 V at the scan rate of 100 mV/s.

### Synthesis of Ag NR

To prepare Ag NRs, the first colloidal silver seed solution was synthesized according to the method described by Creighton et al.^70,71^ with some modifications. In brief, 2 mL of 0.01 M AgNO_3_, 0.2 mL of 0.1 M CTAB, and 2 mL of 0.01 M sodium citrate were mixed, and 0.6 mL of 0.01 M NaOH was added. Then, deionized water was added to obtain a total volume of 20 ml and stirred vigorously for one minute. Then, 0.6 mL of 10 mM solution of ice-cold NaBH4 was added to the mixed solution and stirred until the solution color changed from bright yellow to green, indicating the formation of silver nanoparticles. This product is the seed solution that was used to synthesize Ag NR.

For synthesizing Ag NR, 0.25 ml of 10 mM AgNO_3_, 0.50 ml of 100 mM ascorbic acid, and 10 ml CTAB solution were mixed. Afterward, 0.25 ml of synthesized seed solution and 0.10 ml of 1 M NaOH solution were added to the mixture^71,72^. After several minutes, a change in color to orange-brown took place, indicating the successful formation of Ag NRs. Then, the mixture was centrifuged and stored at room temperature.

### Synthesis of Ag NR@PEI-Ag nanohybrid

To prepare the Ag NR@PEI nanohybrid, 0.2 g of polyethyleneimine (PEI) was dissolved in 1 mL of deionized water and added to the synthesized Ag NRs under stirring. After 12 h of incubation at room temperature, 10 mM AgNO_3_ was added to the synthesized Ag NR@PEI nanohybrid under stirring. After 10 min, the mixture color changed from orange-brown to green-gray, showing Ag NR@PEI-Ag nanohybrid formation.

### Aptamer immobilization and HER2 measurement

To covalently bind the HER2 aptamer onto the electrode surface, each aptamer molecule has a thiol group at the 5' terminus. Before modification, the Au electrodes were polished with alumina slurry. Then, Au electrodes were electrochemically cleaned by cycling between 1.5 and -0.4 V in 0.5 M sulfuric acid (⁓ 15 cycles)^73^. After rinsing the Au electrode with deionized water, to attach the thiolated aptamer onto the electrode surface, 2 µL of aptamer and 2 µL of 0.1 M phosphate-buffered saline (PBS) were mixed and cast on the Au electrode. After drying under an infrared lamp for several minutes, the electrode was washed three times with PBS. Next, HER2 solution was cast on the modified Au electrode to bind to the immobilized aptamer. The electrode was then dried under the infrared lamp and washed with PBS. Finally, the Ag NR@PEI-Ag nanohybrid was cast on the surface of the Au electrode to be adsorbed on the aptamer-HER2 complex. At each step after drying and washing, the CV of the modified Au electrode was recorded in 2 mL of 0.1 M PBS at the potential range of − 0.6 to + 0.6 V, and the scan rate was 100 mV s^−1^. This procedure is summarized in Fig. 4.

## Data Availability

The datasets used and/or analyzed during the current study are available from the corresponding author.
